# The Diseases Admitted as in and out Patients at the Westminster Hospital, between Jan. 20, and Feb. 20, 1799

**Published:** 1799-03

**Authors:** 


### MONTHLY REPORTS OF DISEASES.

*The Diseases admitted as in and out Patients at the Westminster Hospital, between Jan. 20, and Feb. 20, 1799.*

| | | | |
|---|---|---|---|
| ACUTE DISEASES. | | Cephalæa | 2 |
| Bilious Fever | 4 | Chronic Rheumatism | 7 |
| Typhus | 2 | Colic | 2 |
| Pneumonia | 3 | Diarrhœa | 4 |
| Acute Rheumatism | 2 | Dyspepsia | 2 |
| CHRONIC DISEASES. | | Dysuria | 2 |
| Amenorrhœa | 2 | Enterodynia | 2 |
| Anasarca | 3 | Hooping Cough | 4 |
| Ascites | 2 | Hysteria | 1 |
| Asthenia | 4 | Hydrocephalus | 1 |
| Catarrh | 3 | Hydrops-ovari | 1 |

Hydro-

| | | | |
|---|---|---|---|
| Hydro-thorax | 1 | Struma | 2 |
| Impetigo | 2 | Worms | 2 |
| Paralysis | 1 | PERIODIC DISEASES. | |
| Phthisis | 4 | Intermittents | 2 |
| Pleurodynia | 1 | | |

